# Clinical landscape of *TP73* structural variants in ATL patients

**DOI:** 10.1038/s41375-023-02059-9

**Published:** 2023-10-20

**Authors:** Hiroaki Hiramatsu, Rui Yokomori, Liu Shengyi, Norio Tanaka, Seiichi Mori, Kazuma Kiyotani, Osamu Gotoh, Shigeru Kusumoto, Nobuaki Nakano, Youko Suehiro, Asahi Ito, Ilseung Choi, Eiichi Ohtsuka, Michihiro Hidaka, Kisato Nosaka, Makoto Yoshimitsu, Yoshitaka Imaizumi, Shinsuke Iida, Atae Utsunomiya, Tetsuo Noda, Hiroyoshi Nishikawa, Ryuzo Ueda, Takaomi Sanda, Takashi Ishida

**Affiliations:** 1 Department of Immunology, Nagoya University Graduate School of Medicine, Nagoya, Japan; 2 Cancer Science Institute of Singapore, National University of Singapore, Singapore, Singapore; 3 Project for Development of Innovative Research on Cancer Therapeutics, Cancer Precision Medicine Center, Japanese Foundation for Cancer Research, Tokyo, Japan; 4 Project for Immunogenomics, Cancer Precision Medicine Center, Japanese Foundation for Cancer Research, Tokyo, Japan; 5 Department of Hematology and Oncology, Nagoya City University Graduate School of Medical Sciences, Nagoya, Japan; 6 Department of Hematology, Imamura General Hospital, Kagoshima, Japan; 7 Department of Hematology, National Hospital Organization Kyushu Cancer Centre, Fukuoka, Japan; 8 Department of Cell Therapy, National Hospital Organization Kyushu Cancer Centre, Fukuoka, Japan; 9 Department of Hematology, Oita Prefectural Hospital, Oita, Japan; 10 Department of Hematology, National Hospital Organization Kumamoto Medical Center, Kumamoto, Japan; 11 Department of Hematology, Kumamoto University Hospital, Kumamoto, Japan; 12 Department of Hematology and Rheumatology, Kagoshima University Graduate School of Medical and Dental Sciences, Kagoshima, Japan; 13 Department of Hematology, Nagasaki University Hospital, Nagasakin, Japan; 14 Cancer Institute, Japanese Foundation for Cancer Research, Tokyo, Japan; 15 Division of Cancer Immunology, Research Institute/Exploratory Oncology Research and Clinical Trial Center, National Cancer Center, Tokyo, Japan; 16 Department of Medicine, Yong Loo Lin School of Medicine, National University of Singapore, Singapore, Singapore

**Keywords:** Haematological cancer, Lymphoma, T-cell lymphoma

## To the Editor:

*TP73*, a *TP53* family member, is involved in the pathogenesis of adult T-cell leukemia/lymphoma (ATL) and is regulated by the intragenic super-enhancer [1, 2]. We have reported that *TP73* structural variants (SVs) with deletion of exons 2–3, which were potentially associated with the formation of super-enhancer, were present in a fraction of ATL patients. Experimental deletion of exons 2–3 conferred a competitive advantage to ATL cells by promoting their proliferation [2]. Hence, following this observation, here we explored the clinical landscape of *TP73* SVs in ATL patients.

Study subjects were mogamulizumab-naïve ATL patients without prior allogeneic hematopoietic stem cell transplantation, who then received mogamulizumab-containing treatment (*n* = 63) [3]. They had all been enrolled in our previous study [4]. DNA/RNA preparation for genomic analysis, exome library preparation and sequencing, RNA-sequencing, somatic variant call, fusion gene detection using RNA-sequencing, detection of SVs in the 3′-UTR of *CD274* gene, HLA alteration call, and statistical analyses were all as previously described [4]. Tumor purity for tumor-derived DNA was calculated by Control-FREEC [5]. *TP73* SVs, of exons 2 or 2–3 deletion, were evaluated by three different programs, such as DeviCNV v1.5.1 (Rhelixa Inc., Tokyo, Japan) [6], Manta v1.6.0, and GRIDSS2 v2.13.2, using our previous exome dataset [4]. The presence of *TP73* SVs was defined when they were detected by at least one of these three programs. In the cases where *TP73* SVs were identified by any of these programs, the original sequence data were manually investigated to confirm the presence of *TP73* SVs. The current study was approved by the institutional review boards at all participating sites, and all patients provided written informed consent before blood or tissue sampling.

As a result, patient 1, 2, and 3 were determined to harbor exon 2 deletion, and patients 5, and 7 were determined to harbor exons 2–3 deletion, by DeviCNV (Supplementary Table S1). Although, in these five cases, *TP73* SVs were not detected by Manta or GRIDSS2 (Supplementary Table S2 or S3, respectively), the presence of *TP73* SVs was confirmed manually (Supplementary Fig. S1A, S1B, S1C, S1E, and S1G). The discrepancy in result is likely due to the difference in detection methods (e.g., exon-level detection vs breakpoint detection). 　Patient 4 was determined to harbor *TP73* SVs by all three programs, which was confirmed manually (Supplementary Fig. S1D). Patient 6 was determined to harbor exons 2–3 deletion by DeviCNV, and the deletion included a part of exon 4 by both Manta and GRIDSS2. The manual investigation determined this case to harbor *TP73* SVs (Supplementary Fig. S1F). Although patient 8 was not determined to harbor *TP73* SVs by DeviCNV, it was determined by both Manta and GRIDSS2, which was manually confirmed (Supplementary Fig. S2). Patients 9 and 10 were determined to harbor only *TP73* exon 3 deletion by DeviCNV. The manual investigation confirmed these both two to harbor only a part of exon3 deletion (patients 9 and 10, Supplementary Figs. S3 and S4, respectively), thus these were not included in the *TP73* SVs (+) group. Collectively, *TP73* SVs were found in 8 patients (13%), namely patients 1–8.

**Fig. 1:**
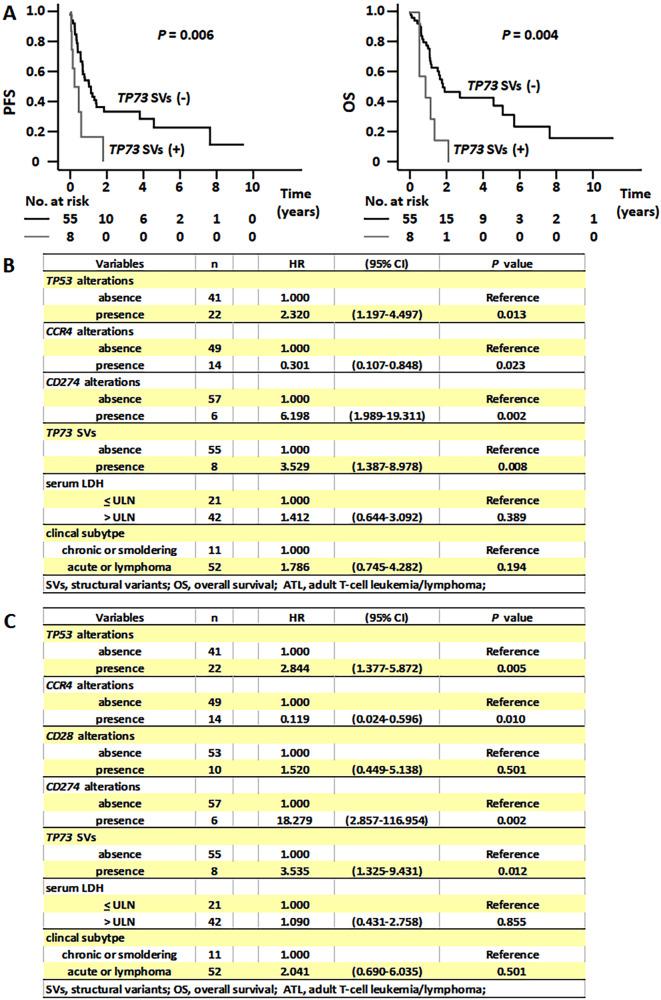
Prognostic significance of *TP73* SVs in patients with adult T-cell leukemia/lymphoma. **A** Progression-free survival (PFS) of adult T-cell leukemia/lymphoma (ATL) patients with or without *TP73* SVs (left panel). Overall survival (OS) of ATL patients with or without *TP73* SVs (right panel). **B** Multivariate analysis including *TP73* SVs for PFS in patients with ATL. **C** Multivariate analysis including *TP73* SVs for OS in patients with ATL.

As for clinical characteristics, there were no significant differences between patients with or without *TP73* SVs in terms of age, sex, previous systemic chemotherapy, Eastern Cooperative Oncology Group performance status or clinical subtype. On the other hand, serum LDH was significantly higher in patients with SVs than in those without (Supplementary Table S4). Notably, *TP73* SVs held prognostic significance: median progression-free survival (PFS) of patients with *TP73* SVs was 0.2 years versus 1.1 years for those without (*P* = 0.006; Fig. 1A, left panel). In addition, the median overall survival (OS) of the patients with or without *TP73* SVs was 0.8 and 1.8 years, respectively (*P* = 0.004; Fig. 1A, right panel). Then, we performed multivariate analysis of PFS of the 63 ATL patients using six variables as follows: *TP73* SVs, *TP53*, *CCR4,* and *CD274* alterations, or clinical subtype and serum LDH. These three gene alterations were found to be significantly associated with PFS in our previous univariate analysis [4], and these two clinical variables were generally acceptable prognostic factors in ATL [7]. As a result, *TP73* SVs were significantly associated with PFS (Fig. 1B). Multivariate analysis also for OS was performed using seven variables, such as *TP73* SVs, and *TP53*, *CCR4*, *CD28*, and *CD274* alterations, because the latter four gene alterations were significantly associated with OS previously in univariate analysis [4], or clinical subtype or serum LDH. Eventually, *TP73* SVs were also significantly associated with OS (Fig. 1C). Collectively, these indicate that *TP73* SVs are significantly and independently associated with a worse prognosis for ATL patients.

Next, we sought relationships between the presence of *TP73* SVs and the 34 ATL driver gene alterations that were found in more than two patients each (Supplementary Table S5) [4]. This analysis revealed that patients with *TP73* SVs significantly more frequently harbored *TBL1XR1, or RHOA* alterations, but there were no significant differences in the remaining 32 genes (Supplementary Table S6).

**Fig. 2:**
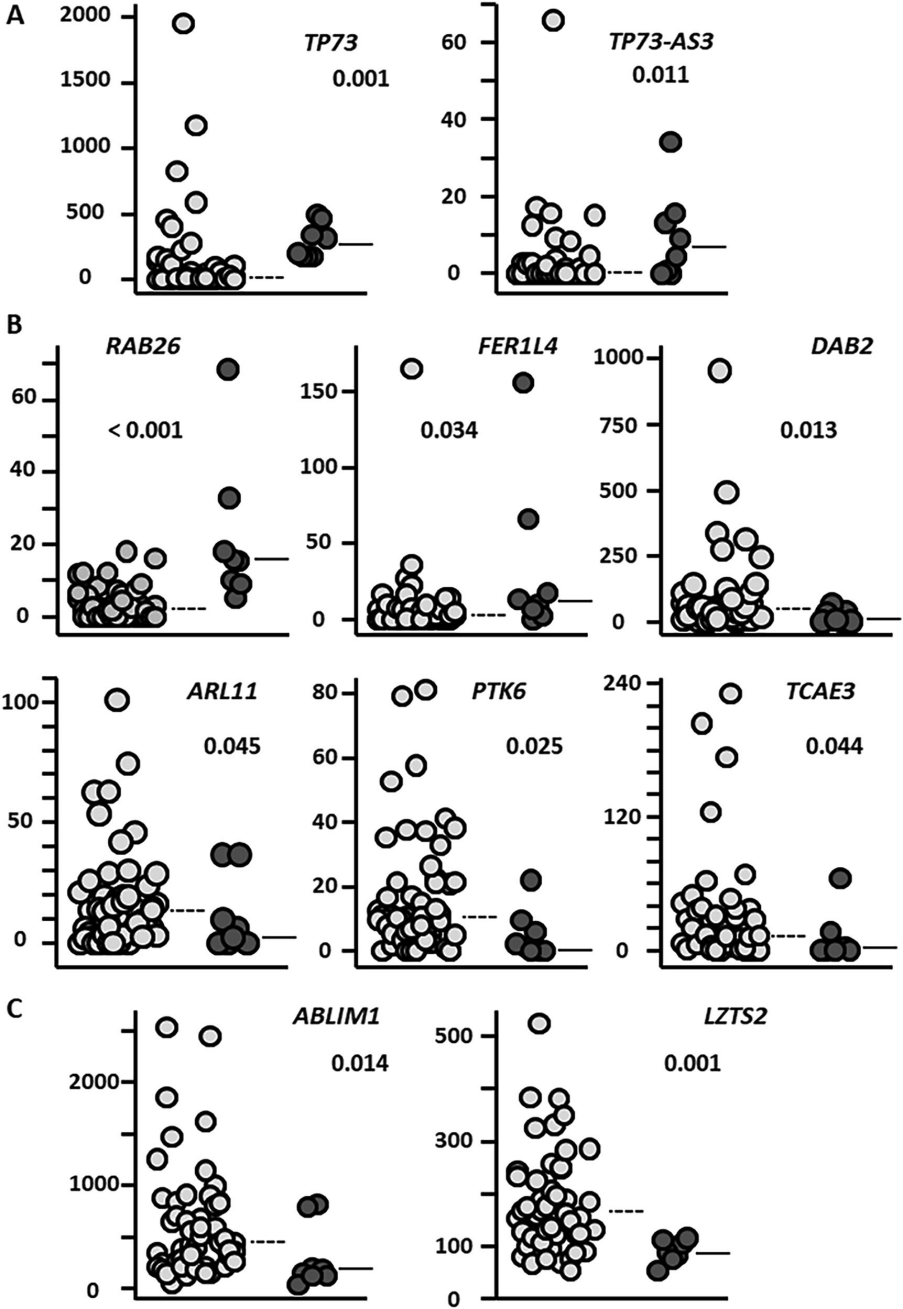
Transcriptome profiles according to *TP73* SVs. **A** *TP73* expression is higher in patients with *TP73* SVs (dark gray circles, right side, *n* = 8) than in those without (light gray circles, left side, *n* = 55) (median, 260.1 vs. 13.8 TMM [trimmed mean of *M* values], *P* = 0.001 [Mann–Whitney *U* test]) (upper left panel). *TP73-AS3* expression is higher in patients with *TP73* SVs than in those without (median, 6.8 vs. 0 TMM, *P* = 0.011) (upper right panel). **B** *RAB26* expression is higher in patients with *TP73* SVs (dark gray circles, right side) than in those without (light gray circles, left side) (median, 15.5 vs. 2.1 TMM, respectively, *P* < 0.001) (upper left panel). *FER1L4* expression is also higher in patients with *TP73* SVs (median, 11.8 vs. 2.8 TMM, respectively *P* = 0.034) (upper middle panel). *DAB2* expression is lower in patients with *TP73* SVs compared to those without (median, 7.8 vs. 45.8 TMM, *P* = 0.013) (upper right panel), as is *ARL11* expression (2.3 vs. 13.3 TMM, *P* = 0.045) (lower left panel), *PTK6* expression (1.8 vs. 10.3 TMM, *P* = 0.025) (lower middle panel), and *TCAE3* expression (1.3 vs. 12.6 TMM, *P* = 0.044) (lower right panel). The horizontal straight and dotted lines represent the median value of gene expression (TMM, trimmed mean of M-values) in patients with and without *TP73* SVs, respectively. **C** *ABLIM1* expression is lower in patients with *TP73* SV than in those without (median, 170.1 vs. 451.1 TMM, *P* = 0.014) (left panel). Also, *LZTS2* expression is lower in patients with *TP73* SVs compared to those without (median 87.1 vs. 149.3 TMM, *P* = 0.001).

Using RNA-sequencing, we then analyzed the transcriptome profiles in ATL stratified according to *TP73* SVs (cBioinformatics Inc., Tokyo, Japan). First, we confirmed that there were no significant differences in tumor purity between two groups (76.3% [median], and 57.2–99.1% [range] in eight patients with SVs, and 86.6%, and 52.9–100.0%, in 55 patients without SVs, *P* = 0.137). We previously reported that consensus clustering analysis of the present patients defines four transcriptome subtypes (TS) designated A, B, C, and D [4]. Here, we found no significant associations of *TP73* SVs with a particular TS: A (1/19), B (3/18), C (0/5), and D (4/21) (*P* = 0.551). We then performed individual gene expression analysis, which revealed that *TP73* expression was higher in patients with *TP73* SVs than in those without (Fig. 2A, left panel). Similarly, the expression of *TP73-AS3* (NCBI/Entrez Gene ID: 105378610), which is located on the anti-sense strand within the intragenic super-enhancer region of *TP73*, was higher in patients with SVs (Fig. 2A, right panel) (Supplementary Table S6). This finding is consistent with our previous report that *TP73* SVs were associated with the activation status of *TP73* gene transcription [2].

Next, of the 228,048 transcripts in GENCODE Release 34, we selected 28 genes which were upregulated or 60 downregulated in ATL cells with *TP73* SVs, compared to those without SVs, based on our pre-determined criteria (Supplementary Table S8). From this selection, six notable findings emerged, namely, 1) *RAB26* expression was higher in patients with *TP73* SVs than in those without (Fig. 2B, upper left panel). This is consistent with the fact that *RAB26* contributes to the progression of non-small cell lung cancer [8]. 2) A long non-coding RNA, *FER1L4*, expression was higher in patients with SVs (Fig. 2B, upper middle panel), consistent with the fact that *FER1L4* is the direct transcriptional target of *TP73* [9]. In contrast, expressions of 3) *DAB2* [10], 4) *ARL11* [11], 5) *PTK6* [12], and 6) *TCAE3* [13], were lower in patients with SVs (Fig. 2B, upper right, lower left, lower middle, and lower right panels, respectively). These four genes have been reported to be tumor suppressors, and especially *DAB2* is associated with c-Myc downregulation [10].

Other than these selected genes, we also paid special attention to those genes that were regulated by experimental deletion of *TP73* exons 2–3 in an ATL cell line (TL-Om1) in our previous study [2]. Among those genes, notably *ABLIM1* and *LZTS2* expression was lower in primary ATL cells with SVs than without (Fig. 2C, left and right panels, respectively, and Supplementary Table S6). It has been reported that overexpression of *ABLIM1* in glioblastoma cells leads to an attenuated proliferation [14], and deletion of *LZTS2* increases susceptibility to spontaneous and carcinogen-induced tumor development [15], thus suggesting that downregulation of these genes contributes to the aggressiveness of ATL. Collectively, these gene expression profiles differing between patients with or without *TP73* SVs are consistent with our previous findings in which ATL cells with *TP73* SVs acquired enhanced resistance to apoptosis and a growth advantage [2]. In addition, these gene profiles are also consistent with the present miserable prognosis of ATL patients with *TP73* SVs.

To the best of our knowledge, this is the first report to describe the clinical landscape of *TP73* SVs in patients with ATL, or indeed any type of cancer. Although the present investigation offers significant observations regarding *TP73* SVs, an unavoidable limitation should be recognized. The presence or absence of *TP73* SVs was evaluated using three different programs. Because varying tumor purity of the samples cannot be integrated into this program, despite our confirmation of the lack of significant differences in this respect, it cannot be completely excluded that the occurrence of *TP73* SVs may have been underestimated in the present study.

In conclusion, the presence of *TP73* SVs was significantly and independently associated with a worse prognosis of ATL patients receiving mogamulizumab-containing treatment. ATL cells with *TP73* SVs exhibit gene expression profiles associated with enhanced resistance to apoptosis and growth advantage. Taken together, we believe that the current results contribute to a better understanding of the pathogenesis not only of ATL, but also many types of cancer associated with *TP73*, and particularly with *TP73* SVs.

### Supplementary information

Supplementary Table S1

Supplementary Table S2

Supplementary Table S3

Supplementary Table S4

Supplementary Table S5

Supplementary Table S6

Supplementary Table S7

Supplementary Table S8

Supplementary Figures

## References

[1] Wong RWJ, Ngoc PCT, Leong WZ, Yam AWY, Zhang T, Asamitsu K (2017). Enhancer profiling identifies critical cancer genes and characterizes cell identity in adult T-cell leukemia. Blood..

[2] Ong JZL, Yokomori R, Wong RWJ, Tan TK, Ueda R, Ishida T (2022). Requirement for TP73 and genetic alterations originating from its intragenic super-enhancer in adult T-cell leukemia. Leukemia..

[3] Ishii T, Ishida T, Utsunomiya A, Inagaki A, Yano H, Komatsu H (2010). Defucosylated humanized anti-CCR4 monoclonal antibody KW-0761 as a novel immunotherapeutic agent for adult T-cell leukemia/lymphoma. Clin Cancer Res.

[4] Tanaka N, Mori S, Kiyotani K, Ota Y, Gotoh O, Kusumoto S (2022). Genomic determinants impacting the clinical outcome of mogamulizumab treatment for adult T-cell leukemia/lymphoma. Haematologica..

[5] Boeva V, Popova T, Bleakley K, Chiche P, Cappo J, Schleiermacher G (2012). Control-FREEC: a tool for assessing copy number and allelic content using next-generation sequencing data. Bioinformatics..

[6] Kang Y, Nam SH, Park KS, Kim Y, Kim JW, Lee E (2018). DeviCNV: detection and visualization of exon-level copy number variants in targeted next-generation sequencing data. BMC Bioinform.

[7] Shimoyama M (1991). Diagnostic criteria and classification of clinical subtypes of adult T-cell leukemia-lymphoma. A report from the Lymphoma Study Group (1984–1987). Br J Haematol.

[8] Ren H, Yang B, Li M, Lu C, Li X (2022). RAB26 contributes to the progression of non-small cell lung cancer after being transcriptionally activated by SMAD3. Bioengineered.

[9] Uboveja A, Satija YK, Siraj F, Saluja D (2022). p73-regulated FER1L4 lncRNA sponges the oncogenic potential of miR-1273g-3p and aids in the suppression of colorectal cancer metastasis. iScience.

[10] Li H, Zhou Y, Wang M, Wang H, Zhang Y, Peng R (2021). DOC-2/DAB2 interactive protein destabilizes c-Myc to impair the growth and self-renewal of colon tumor-repopulating cells. Cancer Sci.

[11] Casalou C, Ferreira A, Barral DC (2020). The role of ARF family proteins and their regulators and effectors in cancer progression: a therapeutic perspective. Front Cell Dev Biol.

[12] Liu B, Yao X, Zhang C, Liu Y, Wei L, Huang Q (2023). PTK6 inhibits autophagy to promote uveal melanoma tumorigenesis by binding to SOCS3 and regulating mTOR phosphorylation. Cell Death Dis.

[13] Kazim N, Adhikari A, Oh TJ, Davie J (2020). The transcription elongation factor TCEA3 induces apoptosis in rhabdomyosarcoma. Cell Death Dis.

[14] Liu D, Wang X, Liu Y, Li C, Zhang Z, Lv P (2022). Actin-binding LIM 1 (ABLIM1) inhibits glioblastoma progression and serves as a novel prognostic biomarker. Dis Mark.

[15] Johnson DT, Luong R, Lee SH, Peng Y, Shaltouki A, Lee JT (2013). Deletion of leucine zipper tumor suppressor 2 (Lzts2) increases susceptibility to tumor development. J Biol Chem.

