# Supplementary material for: Clinical landscape of TP73 structural variants in ATL patients
Source: Leukemia. 2023 Oct 20;37(12):2502–6. doi: 10.1038/s41375-023-02059-9 (PMC10681890; doi:10.1038/s41375-023-02059-9)
Supplement: Supplementary file 9 — Supplementary Figures [file 41375_2023_2059_MOESM9_ESM.pdf]

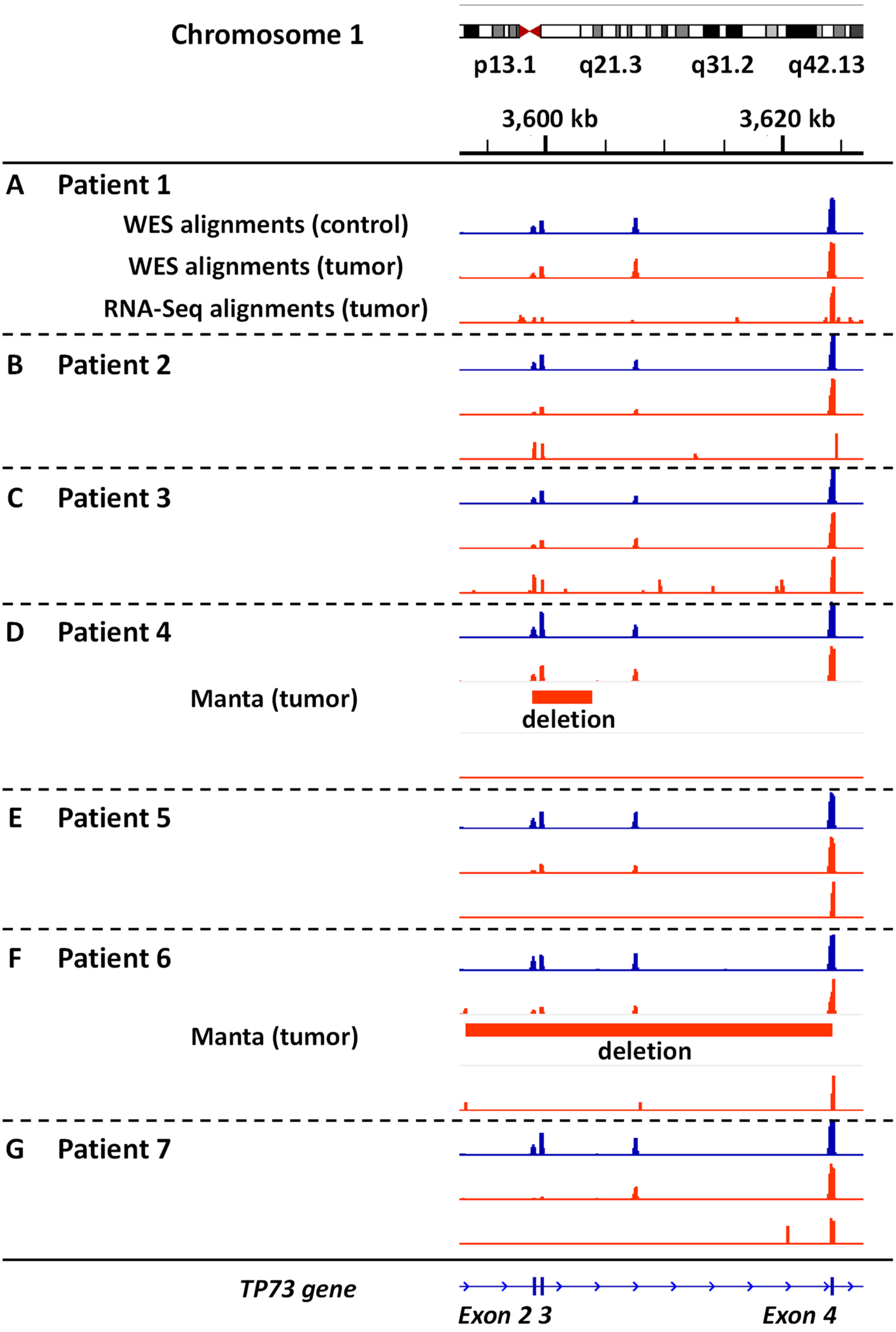
Chromosome 1
p13.1
q21.3
q31.2
q42.13
3,600 kb
3,620 kb
A Patient 1
WES alignments (control)
WES alignments (tumor)
RNA-Seq alignments (tumor)
B Patient 2
C Patient 3
D Patient 4
Manta (tumor)
deletion
E Patient 5
F Patient 6
Manta (tumor)
deletion
G Patient 7
TP73 gene
Exon 2 3
Exon 4

Supplementary Figure S1. Coverage of *TP73* gene. Coverage of *TP73* gene of 7 ATL patients (Patient 1, 2, 3, 4, 5, 6, and 7, for A, B, C, D, E, F, and G, respectively). Exome sequencing of patients' non-tumor control DNA, and tumor DNA were indicated by red (upper rows) and blue (middle rows) lines, respectively. RNA-sequencing of patients' tumors were indicated by red lines (lower rows). Genomic deletions detected by Manta are indicated by red closed squares. WES, whole exome sequencing. RNA-Seq, RNA-sequencing.

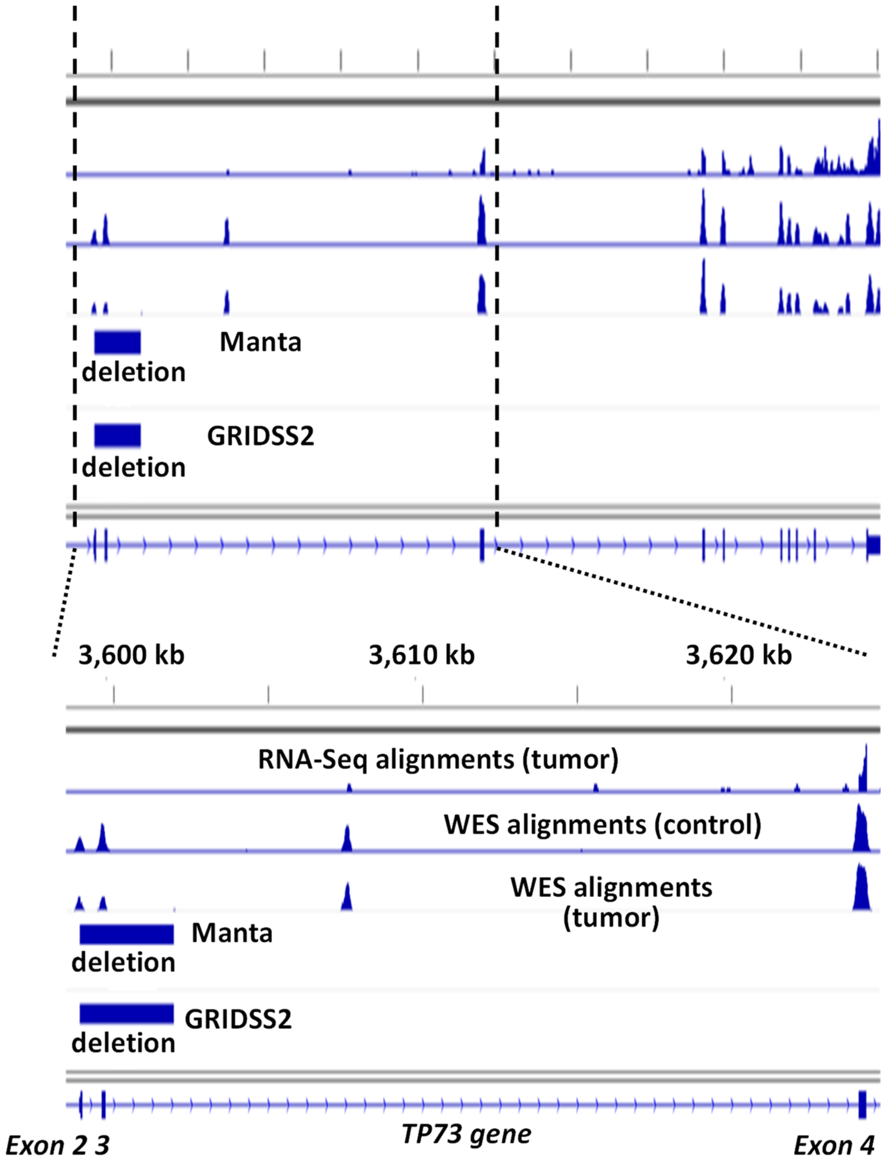

Supplementary Figure S2. Coverage of *TP73* gene of ATL patient 8.

RNA-sequencing of patient's tumor are indicated in upper rows. Exome sequencing of patients' non-tumor control DNA, and tumor DNA are indicated in middle and lower rows, respectively. Genomic deletions detected by Manta and GRIDSS2 are indicated by closed squares. Patient 8 is determined to harbor *TP73* exons 2-3 deletion.

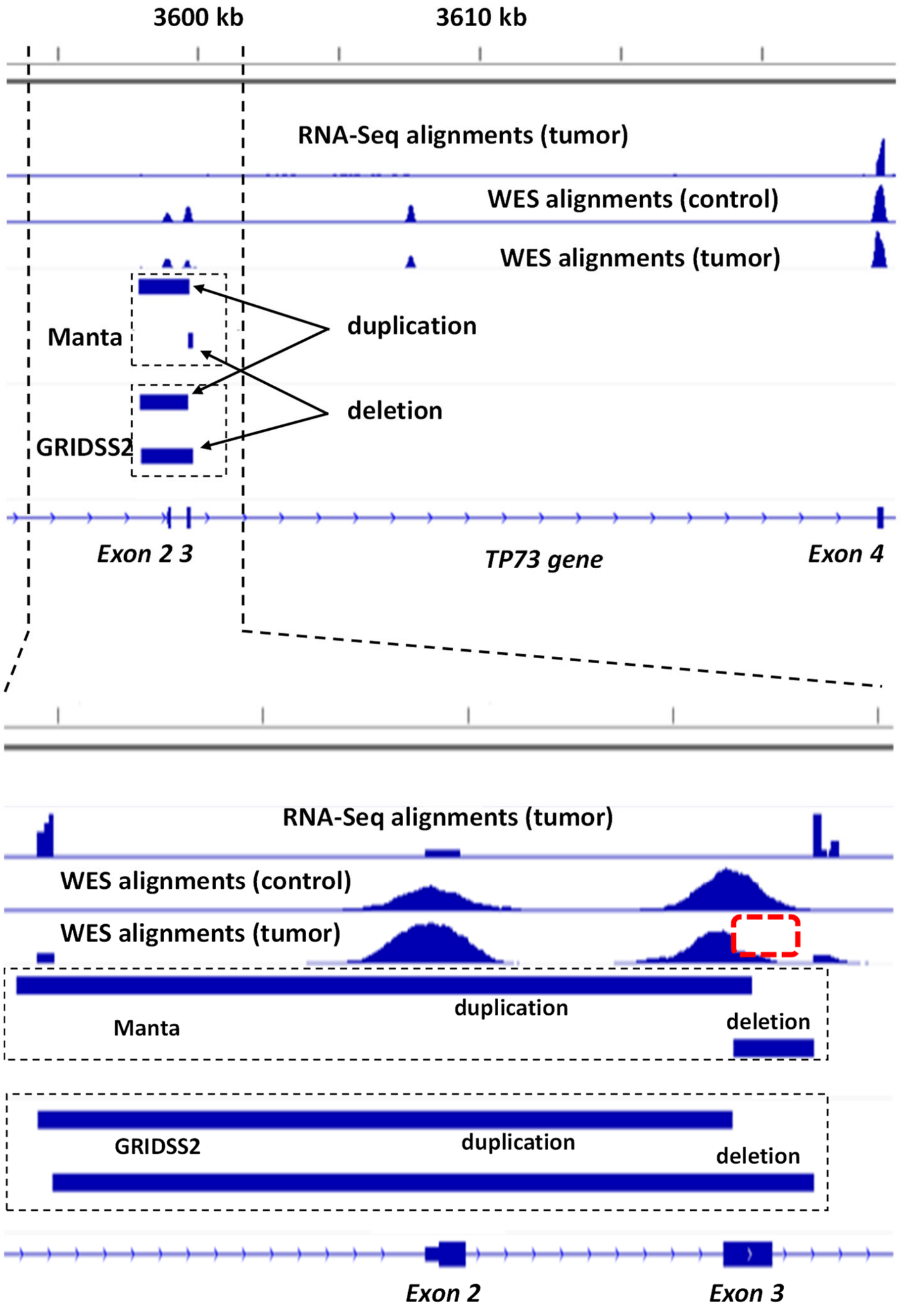

Supplementary Figure S3. Coverage of *TP73* gene of ATL patient 9.

RNA-sequencing of patient's tumor are indicated in upper rows. Exome sequencing of patients' non-tumor control DNA, and tumor DNA are indicated in middle and lower rows, respectively. Genomic deletions detected by Manta and

GRIDSS2 are indicated by closed squares. Patient 9 is determined to harbor a part of *TP73* exon 3 deletion (indicated by red dotted square).

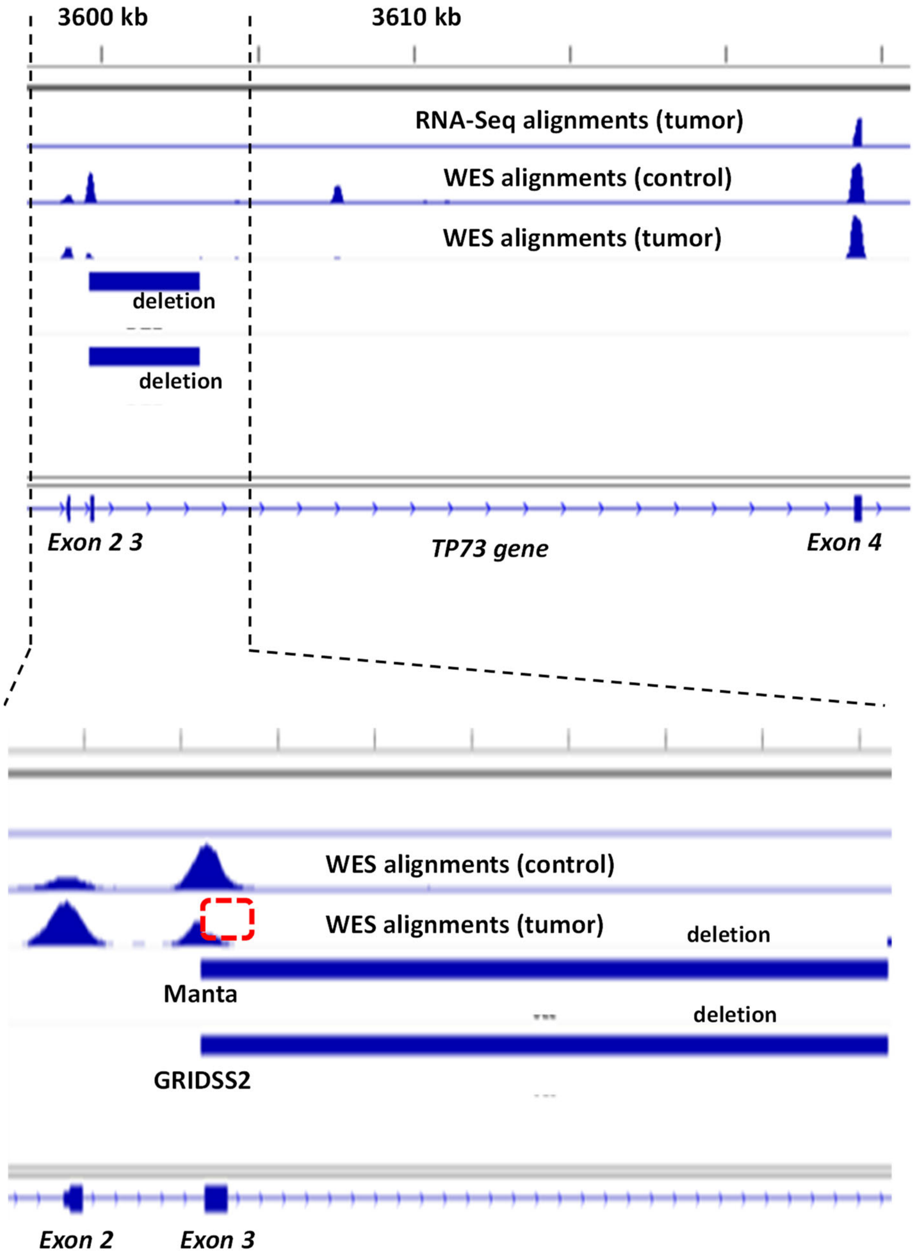

Supplementary Figure S4. Coverage of *TP73* gene of ATL patient 10.

RNA-sequencing of patient's tumor are indicated in upper rows. Exome sequencing of patients' non-tumor control DNA, and tumor DNA are indicated in middle and lower rows, respectively. Genomic deletions detected by Manta and GRIDSS2 are indicated by closed squares. Patient 10 is determined to harbor a part of *TP73* exon 3 deletion (indicated by red dotted square).
